# Rye Bread Crust as an Inducer of Antioxidant Genes and Suppressor of NF-κB Pathway In Vivo

**DOI:** 10.3390/nu14224790

**Published:** 2022-11-12

**Authors:** Kristin Wächter, Birte Gohde, Gábor Szabó, Andreas Simm

**Affiliations:** 1 Department for Cardiac Surgery, University Hospital Halle (Saale), Martin-Luther University, Halle-Wittenberg, 06120 Halle (Saale), Germany; 2 Center for Medical Basic Research, Martin-Luther-University Halle-Wittenberg, 06120 Halle (Saale), Germany

**Keywords:** advanced glycation end products, bread crust, NRF2, in vivo imaging, NF-κB-luciferase-reporter mice, cardioprotection, antioxidant

## Abstract

Heat-processed food, like bread, containing high amounts of advanced glycation end products (AGEs), is controversially discussed regarding the effects on health and disease. In in vitro and in vivo experiments, AGEs can induce proinflammatory NF-κB and/or the anti-inflammatory NRF2 pathways. The aim of this study was to investigate how gene expression is influenced in vivo upon short as well as long-term feeding of mice with control and bread crust-food (BC). For that, the liver, kidney and heart from two days- and eight days-fed mice were isolated and gene arrays were performed. Fewer genes were affected in terms of expression after two days of BC feeding than after eight days. We observed, especially in the heart and to lesser extent in the liver, an induction of antioxidant response by BC. Among the significantly up-regulated genes identified in the heart were transcripts encoding for cardioprotective and antioxidative proteins like metallothionein 2, uncoupling protein 3 and pyruvate dehydrogenase kinase 4. In contrast, in the liver, genes encoding for inflammatory drivers like thioredoxin-interacting protein, lncRNA Mtss1 and ubiquitin-specific protease 2 were down-modulated. However, an increased expression of immunoglobulins was observed in the kidney. Furthermore, in vivo imaging analyses with NF-κB-luciferase-reporter mice uncovered a rather anti-inflammatory response, especially after three and seven days of the feeding study. Our results suggest that bread crust exerts antioxidant and anti-inflammatory effects in the model organism mouse in an organ-specific manner.

## 1. Introduction

AGEs (advanced glycation end products) are non-enzymatic protein modifications that play an essential role in aging and degenerative diseases [1]. They are developed by non-enzymatic reactions of protein amino group with reducing sugar [2]. Whereas endogenously produced AGEs induce diseases and aging, the discussion around AGE-rich foods is more controversial. Bread crust (BC) represents such an example of an AGE-rich food. Ingestion of pure AGE-modified proteins like AGE-modified albumin is considered to be rather unhealthy, while for BC many positive effects are known. In 2002, Lindenmeier and colleagues found that BC mediates an antioxidant and chemopreventive activity [3]. Somoza’s research group [4] found an increased antioxidant status in the blood plasma of BC-fed rats, as well as the up-regulation of chemopreventive enzymes (NADPH-cytochrome C reductase, glutathione S-transferase) in the liver. Furthermore, it was shown that extracts from bread crust protect mouse cardiac fibroblasts from oxidative stress and the antioxidant enzyme manganese superoxide dismutase is more strongly expressed [5]. Our group has shown that feeding BC to mice inhibits the growth or migration of lung tumor cells [6]. In addition, a positive effect was observed on the enzymatic defense of the liver (increased formation of catalase and glutathione peroxidase) after BC feeding of rats [7]. Recently, we showed through in vitro experiments that extracts from bread crusts (BCE) induce the nuclear factor erythroid 2-related factor 2 (NRF2)-dependent antioxidant gene expression, thus provoking a protection mechanism against oxidative stress-mediated tissue injury [8]. In line with these findings, we found that BCE protects rat vascular grafts from in vitro ischemia/reperfusion injury, presumably by decreasing nitro-oxidative stress-induced cell death and by up-regulation of the antioxidant gene Gclc [9]. Since absorption of AGE’s by the intestinal tract is quite low, we studied short- vs. long-term fed mice (2 vs. 8 days) with BC- vs. control-food by gene expression analyses (gene arrays) of different isolated organs. Furthermore, in vivo imaging analyses with NF-κB reporter mice, which respond to NF-κB activation by synthesis of the reporter protein firefly luciferase, uncovered a rather anti-inflammatory response especially after three and seven days of the feeding study.

## 2. Materials and Methods

### 2.1. Animal Care

Transgenic, heterozygous mice (B10.Cg-*H2^k^* Tg(NFkB/Fos-luc)26Rinc/J; Jackson Laboratory, catalog number 006100), expressing the firefly luciferase reporter gene under the control of NF-κB regulatory elements, were bred in the center for medical basic research (ZMG) of the Martin Luther University Halle-Wittenberg, Germany, under controlled and sterile conditions (12 h day/night cycle, 24 °C). All animal treatments were approved by the local Animal Care Committee of Sachsen-Anhalt, Germany (approval code: 203.42502-2-1611 MLU). 4–8-month-old mice were used in this study. Mice of the control group received standard diet (70% (*w*/*w*) protein-free standard chow [C1004; Altromin, Lage, Germany], 15% casein, 15% starch) and mice of the BC group received a bread crust-supplemented food (70% (*w*/*w*) protein-free standard chow, 15% casein, 15% rye bread crust) and had unlimited access to food and water. Food intake and mouse weight were controlled continuously. For in vivo imaging, D-Luciferin (D-Luciferin, potassium salt, AAT Bioquest^®^, Inc., CA, USA; 150 mg/kg body weight; in sterile PBS) was intraperitoneally injected (i.p.) and mice were short-time anesthetized using isoflurane (Forane^®^, Abbott, Wiesbaden, Germany) to remove hair from abdomen with an electrical shaver (Contura; Wella Professionals). 15 min after Luciferin-i.p. injection, in vivo imaging was performed using the IVIS Spectrum In Vivo Imaging System (Perkin Elmer) under isoflurane anesthesia, before feeding and at the indicated time points. After two and eight days of feeding, mice were killed by cervical dislocation, and liver, kidney and heart were removed, frozen, and stored at −80 °C. 

### 2.2. Bread Crust

Crust from commercially available whole grain rye bread (KornLiebchen, Halle (Saale), Germany; baking conditions (Table 1)) was isolated from the bread by a 4-edge grater (GEFU, Eslohe, Germany), ground in a mill at 8000 rpm (Retsch, ZM200, Verder Scientific) and frozen at −20 °C. 

### 2.3. RNA Extraction

Total RNA was isolated from organs (liver, kidney, heart) by TRIzol extraction. For this purpose, organs were offset with TRIzol Reagent (Thermo Fisher Scientific, Waltham, MA, USA; 15596018) and homogenized by Tissue Lyser II (Quiagen, Netherlands; 74204). After the addition of chloroform (Sigma Aldrich, MO, USA; 32211-1L-M), mixing and centrifugation (2000× *g*, 5 min) the upper phase was agitated with isopropanol (Sigma Aldrich; MO, USA; 33539-1L-M; 1:1 *v*/*v*), followed by incubation for one hour at room temperature. RNA was pelletized by centrifugation (14,000× *g*, 10 min, 4 °C) and the pellet was washed 3 times with 80% ethanol. After the pellet was dried, nuclease-free water was added and RNA was stored at −20 °C.

### 2.4. Microarray Analyses

An additional RNA purification step was done by using RNeasy MinElute Cleanup Kit (Quiagen, Netherlands; 74204), according to the manufacturer instructions. RNA quality was dissected via use of a Bioanalyzer (2100 Bioanalyzer, Agilent, CA, USA; Supplemental Figure S1A–D). Detection of RNA was performed by microarray, according to the manufacturer’s protocol (CLARIOM D ARRAY, MOU, Thermo Fisher Scientific, Waltham, MA, USA). Shortly, biotin-labeled ss-cDNA was synthesized from total RNA with a GeneChipTM WT PLUS Reagent Kit (Thermo Fisher Scientific, Waltham, MA, USA), and subsequently hybridized using Clariom D mouse arrays (Thermo Fisher Scientific, MA, USA) and GeneChip Fluidics station 450 (Thermo Fisher Scientific, Waltham, MA, USA). Hybridized mRNA chips were washed and scanned by the Affymetrix GeneChip Scanner 7G (GeneChip Command Console 3.1 software). Data validation was performed with a Transcriptome Analysis console (TAC 4.0; applied biosystems; Thermo Fisher Scientific, Waltham, MA, USA). Differentially expressed genes were displayed through fold change (up-regulated > 2.0; down-regulated < −2.0), together with a *p* value < 0.05. Functional annotation of the genes was done with the Consensus Path DB-mouse (cpdb.molgen.mpg.de; MM11; [10]) to find enriched pathways.

## 3. Results

### 3.1. Differentially Expressed RNAs of Control- vs. BC-Fed Mice

#### 3.1.1. Food Intake and Animal Weight

**Figure 1:**
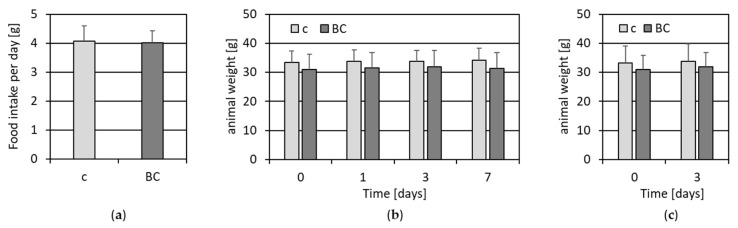
Food intake and animal weight. (**a**) The mean food intake of all animals per day in [g] with standard deviation is shown. Animals received either control food (c) or food supplemented with 15 % bread crust (BC). (**b**) The mean value of animal weight with standard deviation before (0) and upon 1, 3 and 7 days’ feed (*n* = 18; c = 7; BC = 11). (**c**) The mean value of animal weight with standard deviation before (0) and upon 3 days’ feed (*n* = 31; c = 13; BC = 18).

To study the effects of AGE-rich whole grain rye bread crust in vivo, mice were fed for short- and long-term (two vs. eight days) periods with control and BC-food. Food intake and mouse weight were controlled continuously (Figure 1).

The average food intake per day of control, as well as of BC food, comprised 4 g (Figure 1a). The mean animal weight of the control group before feeding started was around 33 g, and did not change over the feeding time of seven days. The mean animal weight of BC-group before feeding started was, at 31 g, a bit less than that of the control-group and stayed constant (Figure 1b). A second feeding experiment with the duration of three days, together with the three days data of Figure 1b, also showed no significant change in animal weight over feeding time, however the control group and the BC group differed in weight by around 2 g. 

#### 3.1.2. Microarray Analysis

**Figure 2:**
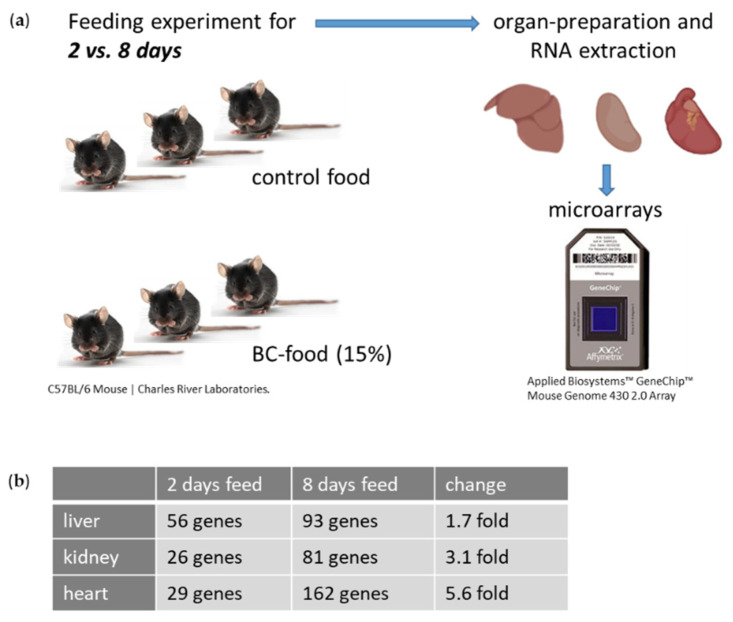
Experimental setup. (**a**) Three animals per food group (control- or BC-fed) were fed for 2 vs. 8 days. Kidney, heart and liver were prepared and after RNA Isolation microarray analyses were performed. (**b**) Overview of differentially expressed genes, which were identified through fold change (up-regulated > 2.0; down-regulated < −2.0) as well as *p*-value < 0.05 (TAC 4.0; applied biosystems; Thermo Fisher Scientific, Waltham, MA, USA). Parts of the figure were created with BioRender.com.

In order to determine whether gene expression is affected in BC- vs. control-fed mice for two vs. eight days, the liver, kidney and heart were removed, RNA was extracted (Supplemental Figure S1A–D) and gene expression was analyzed by Affymetrix array technology (Figure 2a). Differentially expressed RNAs of BC- vs. control-fed mice in the different organs were identified through fold change (up-regulated > 2.0; down-regulated < −2.0) as well as *p*-value < 0.05 (TAC 4.0; applied biosystems; Thermo Fisher Scientific, Waltham, MA, USA) (Figure 2b). As suspected more genes were affected after eight days’ feeding compared to two days’ feeding, however the change between the two feeding durations was more pronounced in heart (Figure 2b; 29 vs. 162 affected genes; 5.6 fold change). 

##### Differentially Expressed RNAs in Liver, Kidney and Heart

**Figure 3:**
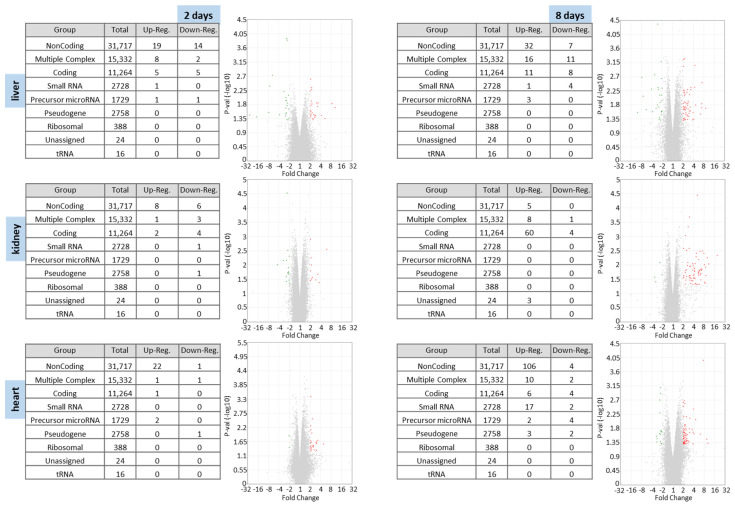
Microarray data: Differentially expressed RNAs of liver, kidney and heart of BC- vs. control-fed mice for 2 and 8 days. Summary tables of the number of differentially expressed RNAs. Cluster analyses of differentially expressed RNAs and corresponding Volcano plot analysis of BC vs. control samples. The *x* axis represents the difference between the two stimulation conditions (regulated genes from −32 to 32 fold change are shown) and the *y* axis represents the −log10 *p* value. Significantly up-regulated RNAs are represented as red dots, and significantly down-regulated RNAs are represented as green dots.

In the next step the number of differentially expressed RNAs in liver, kidney and heart of BC- vs. control-fed mice for two and eight days were summarized (Figure 3). A high number of noncoding RNAs were affected in the bread crust group compared to the control group. The corresponding Volcano plots emphasize an increased number of up-regulated genes compared to down-regulated genes throughout all analyzed organs. 

##### Affected Genes in the Liver of BC- vs. Control-Fed Mice

**Figure 4:**
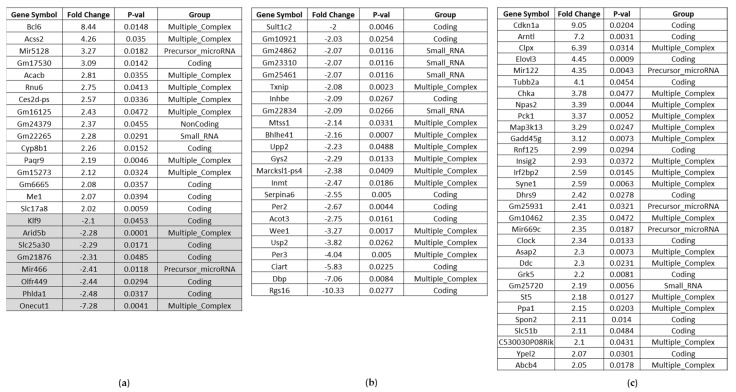
Affected genes in the liver of BC- vs. control-fed mice. (**a**) Significantly up-regulated and down-regulated genes (highlighted in grey) after 2 days BC-fed mice compared to control-fed mice (up-regulated > 2.0; down-regulated < −2.0). (**b**) Significantly down-regulated genes after 8 days BC-fed mice compared to control-fed mice (down-regulated < −2.0). (**c**) Significantly up-regulated genes after 8 days BC-fed mice compared to control-fed mice (up-regulated > 2.0). Noncoding RNAs and pseudogenes are not included.

A thorough analysis of the liver array data upon two days of feeding uncovered three genes regulated by the NRF2 signaling pathway: the bile acid synthesis gene Cyp8b1, the antioxidant malic enzyme1 (Me1) and the acyl-CoA synthetase short-chain family member 2 (Acss2) gene were among the up-regulated genes in the liver. The gene encoding for B cell leukemia/lymphoma 6 (Bcl6) was the most up-regulated one upon BC feeding, which counteracts oxidative stress [11]. Among the down-modulated genes, Phlda1 was identified, a mediator of inflammatory signal transduction [12,13], (Figure 4a). Noticeably more genes were affected in their expression upon feeding mice for eight days with BC food. Functional annotation analyses of regulated genes for identification of enriched pathways uncovered a sizeable number of genes associated with circadian rhythm (Supplemental Figure S2A,B). Among the most up-regulated were Cdkn1a and Arntl, the latter also being known as Bmal1, which has been shown to activate NRF2-mediated antioxidant pathways [14], (Figure 4c). Furthermore, we identified genes involved in the regulation of response to stress, like Rnf125, Map3k13, Clock, Npas2, Syne1 and Gadd45g. For RING finger protein 125 and miR-669c, an anti-inflammatory impact is known [15,16]. In line with these findings, some genes, postulated to promote inflammatory responses, are down-modulated upon eight days BC feeding (lncRNA Mtss1, Usp2, Txnip, Figure 4b).

##### Affected Genes in the Kidney of BC- vs. Control-Fed Mice

**Figure 5:**
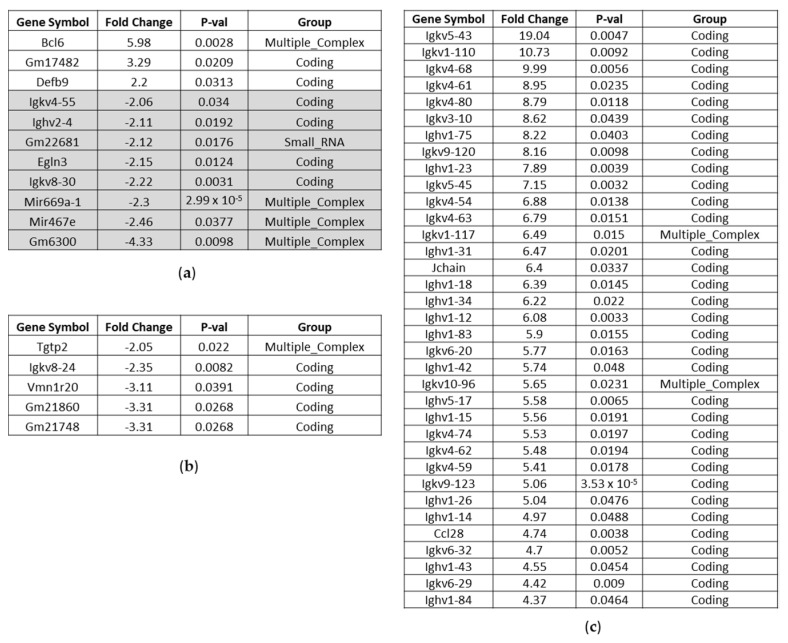
Affected genes in the kidney of BC- vs. control-fed mice. (**a**) Significantly up-regulated and down-regulated genes (highlighted in grey) after 2 days BC-fed mice compared to control-fed mice (up-regulated > 2.0; down-regulated < −2.0). (**b**) Significantly down-regulated genes after 8 days BC-fed mice compared to control-fed mice (down-regulated < −2.0). (**c**) Significantly up-regulated genes after 8 days BC-fed mice compared to control-fed mice (up-regulated ≥ 4.37; up-regulated > 2.0 see Supplemental Figure S3). Noncoding RNAs and pseudogenes are not included.

Upon two days’ feeding just a few genes were affected in kidney from BC-group compared to control-group, however the top up-regulated gene found was also Bcl6 (Figure 5a). In contrast, after eight days BC feeding, we observed a clear shift in immunoglobulin induction compared to control feeding (Figure 5c, Supplemental Figure S3).

##### Affected Genes in the Heart of BC- vs. Control-Fed Mice

**Figure 6:**
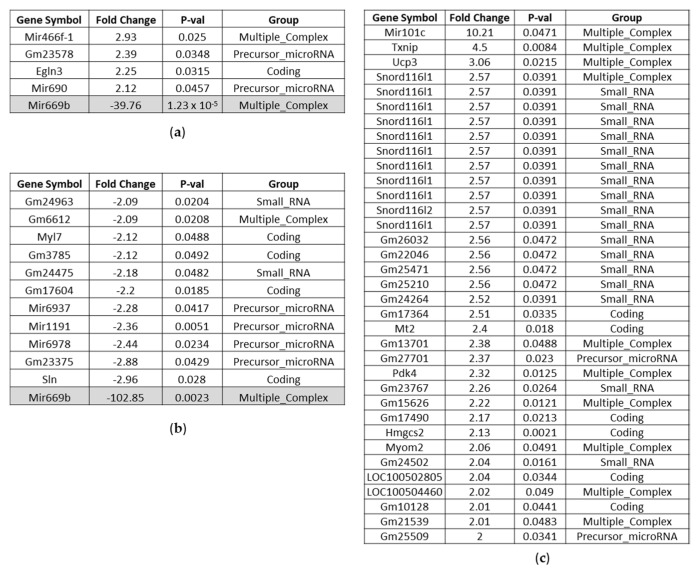
Affected genes in the heart of BC- vs. control-fed mice. (**a**) Significantly up-regulated and down-regulated genes (highlighted in grey) after 2 days BC-fed mice compared to control-fed mice (up-regulated > 2.0; down-regulated < −2.0). (**b**) Significantly down-regulated genes after 8 days BC-fed mice compared to control-fed mice (down-regulated < −2.0). (**c**) Significantly up-regulated genes after 8 days BC-fed mice compared to control-fed mice (up-regulated > 2.0). Noncoding RNAs and pseudogenes are not included.

In the heart gene expression is least affected upon two days of BC- vs. control-fed mice. However, one micro-RNA Mir669b was down-modulated around 40-fold (Figure 6a). Mir669b is associated with inflammatory pathways, and NRF2 is involved in its regulation [17,18]. Among the up-regulated RNAs, Egln3, also known as Phd3, was identified, which is postulated to be cardioprotective [19].

Interestingly Mir669b was even 100-fold down-regulated upon eight days BC feeding compared to control feed (Figure 6b). Among the up-regulated genes, we observed genes like Txnip or Mir101c, which are associated with oxidative stress, but also genes encoding for antioxidant and cardioprotective proteins like metallothionein 2 (Mt2) and uncoupling protein 3 (Ucp3), (Figure 6c). Whereupon Ucp3 is also modulated by NRF2 [20]. Pdk4 (pyruvate dehydrogenase kinase 4) is noteworthy because of its postulated ferroptosis prevention [21].

**Figure 7:**
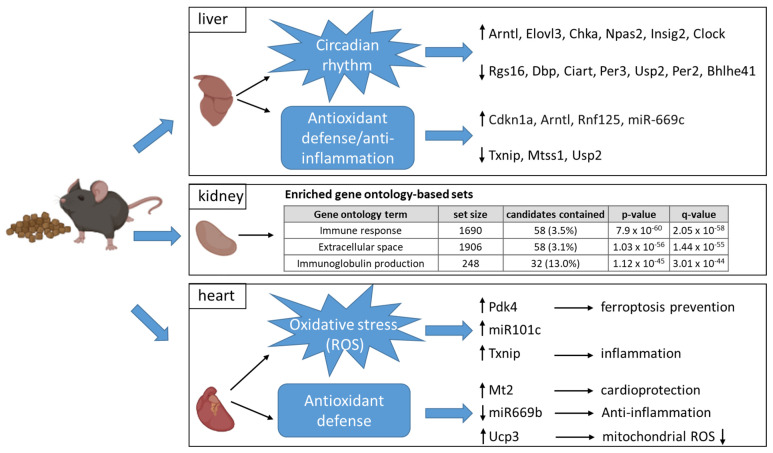
Model of affected genes upon 8 days BC feeding vs. control feeding in liver, kidney and heart. Up-regulated genes are highlighted with black arrows up, and down-regulated genes are highlighted with black arrow down. For kidney functional annotation analyses of up-regulated genes of kidney for the identification of enriched pathways (cpdb.molgen.mpg.de; MM11; [10]). Parts of the figure were created with BioRender.com.

The findings of gene expression analyses from different organs upon eight days BC intervention compared to control intervention, are summarized in Figure 7.

### 3.2. In Vivo Imaging of NF-κB-Luciferase-Reporter Mice

**Figure 8:**
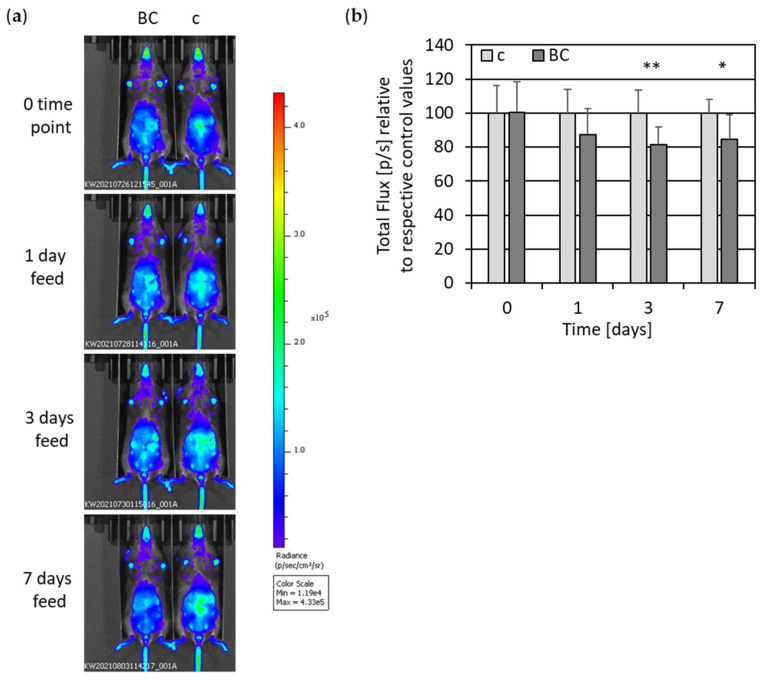
NF-κB induction measured by in vivo luciferase activity of control- vs. BC-fed mice. (**a**) Representative pictures are shown as bioluminescent images with artificial colors in NF-κB-luciferase reporter mice. Bioluminescent images were measured 15 min after i.p. injection of the substrate D-luciferin. In vivo imaging of luciferase activity is shown before (0 time point) and upon 1, 3 and 7 days’ feeding. Animals received either control food (c) or food supplemented with 15 % bread crust (BC). (**b**) Quantification of in vivo imaging of luciferase activity in NF-κB-luciferase reporter mice. Photon emission was determined by IVIS (total Flux [p/s]) and is shown as mean with standard deviation relative to respective control values before (0 time point) and upon 1, 3 and 7 days’ feeding (*n* = 18; c = 7; BC = 11). * *p* ≤ 0.05; ** *p* ≤ 0.01 (*t* test) BCE vs. control-fed mice.

To further explore the anti-inflammatory and antioxidant potential of rye bread crust, we performed in vivo imaging analyses of transgenic reporter mice, expressing firefly luciferase under control of NF-κB regulatory elements. For this purpose, mice were fed with control and BC food, and in vivo imaging was performed before feeding started and at different time intervals during the feeding experiment (Figure 8).

**Figure 9:**
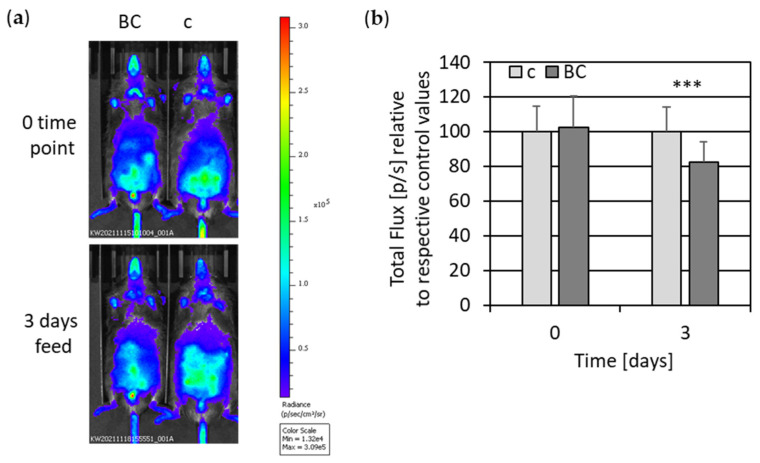
NF-κB induction measured by in vivo luciferase activity of control- vs. BC-fed mice. (**a**) Representative pictures are shown as bioluminescent images with artificial colors in NF-κB-luciferase reporter mice. Bioluminescent images were measured 15 min after i.p. injection of the substrate D-luciferin. In vivo imaging of luciferase activity is shown before (0 time point) and upon 3 days’ feeding. Animals received either control food (c) or food supplemented with 15 % bread crust (BC). (**b**) Quantification of in vivo imaging of luciferase activity in NF-κB-luciferase-reporter mice. Photon emission was determined by IVIS (total Flux [p/s]) and is shown, as a mean with standard deviation relative to respective control values before (0 time point) and upon 3 days’ feeding (*n* = 31; c = 13; BC = 18). *** *p* ≤ 0.001 (*t* test) BCE vs. control-fed mice.

Quantification of all in vivo imaging experiments exhibited a lower activation of the luciferase reporter in BC-fed mice compared to control-fed mice (Figure 8b). Luciferase activity was significantly lower upon three and seven days of BC feeding compared to control feeding. In a second approach, we performed in vivo imaging only upon three days’ intervention, because at this time point change was most significant (Figure 9a). In line with the first in vivo imaging experiment, a pronounced and significant decrease in luciferase reporter activation was monitored in BC-fed mice compared to control-fed mice (Figure 9b).

## 4. Discussion

In the present in vivo study, we provided experimental evidence that feeding mice with bread crust-supplemented food induces antioxidative and cardioprotective gene expression especially, in the heart, and leads to suppression of inflammatory signaling, in part, by decreasing activation of transcription factor NF-κB, up-regulating the antioxidant genes Mt2, Ucp3, Pdk4 and down-modulating miR669b. However, the observed increase in the expression of immunoglobulins in the kidney of mice fed with BC for 8 days compared to control mice, should be considered as critical. We demonstrated that such BC extracts (BCE) can induce the NRF2 pathway in a human endothelial cell line. This leads to protection against oxidative stress-induced cell death [8]. In line with these results, protective effects for arterial grafts in preventing in vitro ischemia/reperfusion injury were shown for extracts from bread crusts [9]. In this study, NRF2 seems to be influenced as well, since NRF2 target genes like Gclc, Nqo1 and Hmox1 are induced upon BCE treatment of aortic rings [9]. Interestingly, metallothionein 2A was also present among up-regulated genes, as identified in the present feeding study, in the heart of BC-fed mice. Metallothioneins (MT) have a cardioprotective potential because of the antioxidant function in the heart [22]. MT acts as a scavenger of ROS like superoxide, hydrogen peroxide, hydroxyl radicals, and nitric oxide [23,24,25]. Additionally, metallothioneins are postulated as anti-inflammatory mediators, and thereby prevent organ injury (reviewed in [26]). Furthermore, transgenic mice overexpressing Mt show inhibited ischemia reperfusion injury in the heart [27]. Duerr and colleagues found a significant increase of Ucp3 expression in the heart of MT^−/−^ mice after one day of I/R, presumably as an auxiliary mechanism to overcome the high load of free radicals [28]. It is noteworthy that both genes, Mt2 and Ucp3, were induced in the mice heart after eight days of BC feeding compared to control feeding. Uncoupling protein 3 is known to be modulated by NRF2 under conditions of oxidative stress [20] and protects tissue against oxidative damage by decreasing the mitochondrial ROS production [29]. Pdk4 was another up-regulated gene identified in the heart upon BC feeding. Ma et al. revealed in 2017 that Nε-carboxymethyl-lysine promotes calcium deposition in vascular smooth muscle cells via oxidative stress-induced PDK4 activation [30]. Nε-carboxymethyl-lysine is a typical advanced glycation end product present in bread crust [8,31,32,33,34,35,36,37]. Recently, PDK4 was demonstrated to prevent ferroptosis by inhibition of pyruvate oxidation and subsequent fatty acid synthesis and lipid peroxidation in pancreatic cancer cells [21]. Ferroptosis is a regulated cell death dependent on iron and accumulation of ROS, which is mitigated by NRF2 [38,39,40,41,42]. NRF2 has been shown to be activated in endothelial cells upon treatment with BCE, a process which leads to the induction of ferroptosis-modulating genes [8]. After eight days BC feeding, we observed a strong down-modulation of miR669b in the heart, an effect which was already visible after two days of BC feeding. Additionally, miR669b is modulated by NRF2 and was identified as a potential biomarker of acute rejection [17,18]. Li and colleagues found, a significant down-regulation of Tnf-α and up-regulation of Il-10 after knocking down miR-669b-3p, which indicates an anti-inflammatory effect [17]. We observed significantly lower luciferase activity in NF-κB reporter mice upon three and seven days’ BC feeding compared to control-fed mice. This can be explained at least partly by the observed change in gene expression upon feeding mice with bread crust. Antioxidant response was also observed upon two days BC feeding. For example, the gene encoding for B cell leukemia/lymphoma 6 (Bcl6) was the most up-regulated gene in the liver and kidney. BCL6 overexpression was shown to inhibit oxidative stress responses to chemotherapeutic reagents in B-cell lymphoma cells [43], and BCL6 knockdown increased hypoxia-induced oxidative stress in cardiomyocytes [44]. Additionally, malic enzyme 1, induced upon two days’ BC feeding in liver, is known to protect against ROS, partly via production of NADPH [45,46]. Consistent with the anti-inflammatory effect observed upon BC feeding, Phlda1, which enhances the activation of TRAF6, thus inducing the NF-κB pathway [12,13], was down-modulated in liver upon 2 days’ BC feeding compared to control feeding. More genes involved in inflammatory response were down-modulated in liver upon eight days’ BC feeding (lncRNA Mtss1, Usp2, Txnip). Txnip was shown to inhibit the antioxidative function of thioredoxin, leading to the accumulation of ROS [47]. USP2 is needed for the phosphorylation of IκB, the nuclear translocation of NF-κB and the expression of NF-κB-dependent target transcripts [48]. Chen and colleagues exhibited that lncRNA Mtss1 promotes inflammatory injury in the brain by increased TRIF expression, P65 phosphorylation, and by enhanced inflammatory cytokine secretion [49]. Among the up-regulated genes upon 8 days’ BC feeding in liver we identified Arntl, which is known to activate NRF2-mediated antioxidant pathways in macrophages [14]. Cdkn1a, the most up-regulated in liver, is thought to be able to interact with NRF2, leading to up-regulation of the NRF2-mediated antioxidant response [50]. Furthermore, Cdkn1a-NRF2 axis was shown to suppress neuroinflammation and thereby rescued neurodegeneration in glial cells [51]. Additionally, the suppression of proinflammatory responses was postulated for the up-regulated miR-669c [16], and for the up-regulated RNF125, a sequential ubiquitination of NLRP3 and thereby limited inflammasome activation was shown [15].

There is also evidence in the literature that heated diets with high levels of glycation end products (AGEs) can have an inflammatory effect and can lead to disadvantages, especially in diseased organisms. For example, renal fibrosis developed in 5/6 nephrectomized rats receiving an AGE diet for a long period of time (13 weeks) [52]. In NOD (non-obese diabetic) mice, ingestion of the AGE-containing diet resulted in ß-cell damage [53]. However, these studies have already been criticized because, overall, these diets were subjected to severe heat treatment, which not only degrades heat-sensitive nutrients but can also result in toxic products [54]. Hence, further studies concerning potential harmful components of bread crust like acrylamide should be undertaken.

In conclusion, bread crust can serve as an inducer of antioxidant defense in vivo, however the beneficial consumption period of bread crust as functional food in humans should be elucidated. This is very important when dietary prevention strategies will be developed.

## Figures and Tables

**Table 1:** Baking conditions.

Grain	Baking Time	Top Heat	Bottom Heat
rye	108 min	250	250

## Data Availability

The data presented in this study are available in the Supplementary Materials. Raw microarray data are available on reasonable request from the corresponding author.

## Supplementary Materials

The following supporting information can be downloaded at: https://www.mdpi.com/article/10.3390/nu14224790/s1, Figure S1: RNA template examination for microarray analyses. Figure S2: Analyses of modulated RNAs in liver upon 8 days’ feeding. Figure S3: Affected genes in the kidney of BC- vs. control-fed mice.

## Abbreviations

AGEs	advanced glycation end products
AVG	average
ARE	antioxidant response element
Arntl	aryl hydrocarbon receptor nuclear translocator-like
BC	bread crust
Bhlhe41	basic helix-loop-helix family, member e41
Bmal1	basic helix-loop-helix ARNT like 1
Cdkn1a	cyclin-dependent kinase inhibitor 1A
Chka	choline kinase alpha
Ciart	circadian associated repressor of transcription
Clock	circadian locomotor output cycles kaput
Cyp8b1	cytochrome P450, family 8, subfamily b, polypeptide 1
Dbp	D site albumin promoter binding protein
Egln3	egl-9 family hypoxia-inducible factor 3
Elovl3	elongation of very long chain fatty acids
Gadd45g	growth arrest and DNA-damage-inducible 45 gamma
Gclc	glutamate-cysteine ligase catalytic subunit
HMOX1	heme oxygenase 1
IL	Interleukin
Insig2	insulin induced gene 2
Map3k13	mitogen-activated protein kinase kinase kinase 13
MNSOD	superoxide dismutase 2
Mtss1	MTSS I-BAR domain containing 1
NADPH	nicotinamide adenine dinucleotide phosphate
NLRP3	NLR family pyrin domain containing 3
Npas2	neuronal PAS domain protein 2
NQO1	NAD(P)H quinone dehydrogenase 1
NRF2	nuclear factor E2-related factor 2
NF-κB	nuclear factor kappa B
PBS	phosphate-buffered saline
Per2, 3	period circadian clock 2
Phlda1	pleckstrin homology like domain, family A, member 1
P65	NF-kB subunit
Rgs16	regulator of G-protein signaling 16
RIN	RNA integrity number
Rnf125	RING finger protein 125
ROS	reactive oxygen species
Syne1	spectrin repeat containing, nuclear envelope protein 1
TNF	tumor necrosis factor a
TRAF6	TNF receptor associated factor 6
TRIF	Toll-like receptor adaptor molecule 1
Txnip	thioredoxin interacting protein
Usp2	ubiquitin specific peptidase 2
