# Supplementary material for: Rye Bread Crust as an Inducer of Antioxidant Genes and Suppressor of NF-κB Pathway In Vivo
Source: Nutrients. 2022 Nov 12;14(22):4790. doi: 10.3390/nu14224790 (PMC9697834; doi:10.3390/nu14224790)
Supplement: Supplementary file 1 [file nutrients-14-04790-s001.zip › nutrients-1991292-supplementary.pdf]

Supplemental data

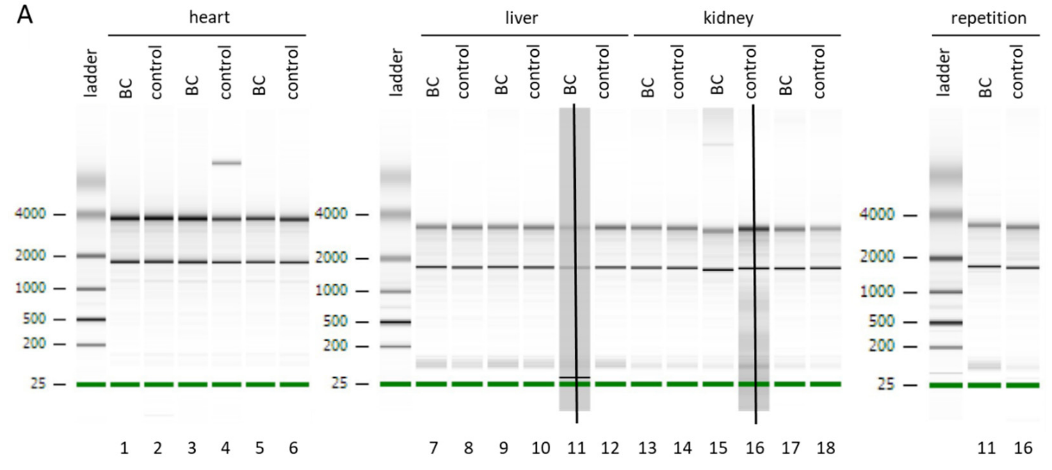

B

sample	RIN value
1	9.1
2	9.0
3	9.2
4	8.9
5	9.0
6	9.0
7	8.0
8	8.6
9	7.9
10	8.4
11	-
12	8.8
13	8.7
14	8.5
15	9.1
16	-
17	8.8
18	8.5
11	8.5
16	8.7

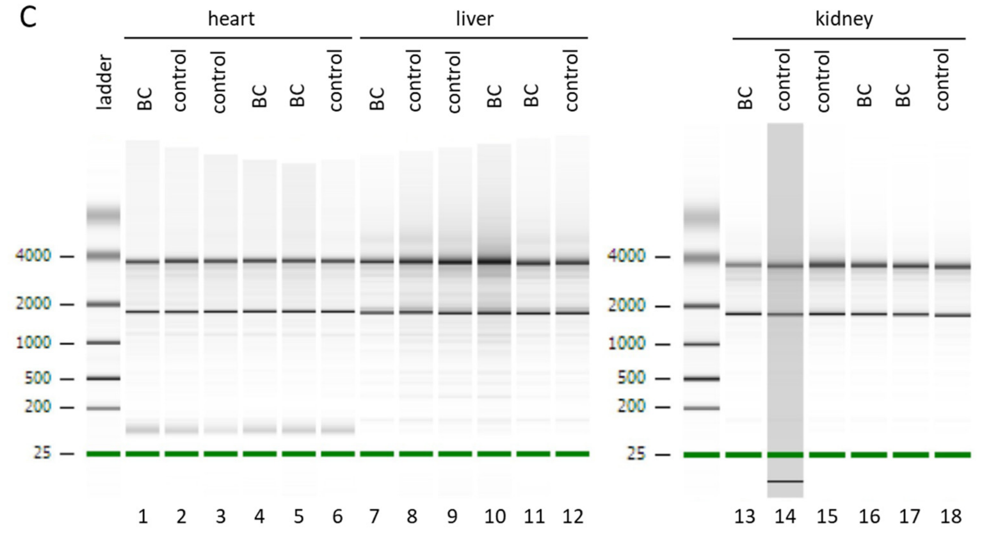

D

sample	RIN value
1	9.1
2	9.1
3	9.4
4	9.4
5	9.2
6	8.7
7	8.6
8	8.4
9	8.2
10	8.1
11	8.7
12	8.3
13	8.9
14	9.2
15	9.4
16	9.2
17	9.0
18	9.3

Figure S1: RNA template examination for microarray analyses. RNA integrity of RNA samples of BC- vs. control-fed mice ((A) 2 days´ feeding, (C) 8 days´ feeding) was verified by Bioanalyzer (2100 Bioanalyzer, Agilent, CA, USA). (B, D) The RNA integrity number (RIN) values of the respective samples.

A **Up-regulated (>2)**

pathway name	set size	candidates contained	p-value	q-value
Circadian rhythm - Mus musculus (mouse)	30	3 (10.0%)	1.55e-05	0.000588
miRNA regulation of DNA Damage Response	82	3 (3.7%)	0.00032	0.00607
FoxO signaling pathway - Mus musculus (mouse)	132	3 (2.3%)	0.00128	0.0127
Dopaminergic synapse - Mus musculus (mouse)	135	3 (2.2%)	0.00134	0.0127
Thyroid cancer - Mus musculus (mouse)	37	2 (5.4%)	0.00168	0.0128
Exercise-induced Circadian Regulation	49	2 (4.1%)	0.00294	0.0165
Endometrial cancer - Mus musculus (mouse)	58	2 (3.4%)	0.00409	0.0165
Basal cell carcinoma - Mus musculus (mouse)	63	2 (3.2%)	0.00481	0.0165
Non-small cell lung cancer - Mus musculus (mouse)	66	2 (3.0%)	0.00527	0.0165
p53 signaling	68	2 (2.9%)	0.00558	0.0165

B **Down-regulated (<-2)**

pathway name	set size	candidates contained	p-value	q-value
Circadian rhythm - Mus musculus (mouse)	30	3 (10.0%)	3.37e-06	2.02e-05
Circadian entrainment - Mus musculus (mouse)	98	2 (2.0%)	0.00437	0.0131
Metapathway biotransformation	141	2 (1.4%)	0.00885	0.0177

Figure S2: Analyses of modulated RNAs in liver upon 8 days' feeding. Functional annotation analyses of up-regulated (A) and down-regulated genes (B), after 8 days BC-fed mice compared to control-fed mice, for the identification of enriched pathways (cpdb.molgen.mpg.de; MM11; [10]).

Gene Symbol	Fold Change	P-val	Group
Ighv6-6	2.04	0.0026	Coding
Ighv1-11	2.08	0.0278	Unassigned
Ighv1-72	2.08	0.0274	Coding
Ighv1-81	2.31	0.0009	Coding
Igkv4-78	2.37	0.0339	Multiple_Complex
Ighv1-79	2.47	0.0259	Unassigned
Igkv14-130	2.49	0.0145	Coding
Xlr3b	2.51	0.0031	Multiple_Complex
Ctxn3	2.61	0.0104	Coding
Iglv2	2.73	0.0082	Multiple_Complex
Ighv1-53	2.95	0.0269	Coding
Ighv1-73	2.97	0.0241	Coding
Igkv14-111	2.97	0.0026	Coding
Ighv1-7	3.03	0.0002	Coding
Igkv4-69	3.11	0.0114	Coding
Igkv4-56	3.11	0.0341	Coding
Igkv4-58	3.17	0.024	Coding
Igkj3	3.19	0.0288	Multiple_Complex
Igkv4-60	3.2	0.0109	Unassigned
Ighv1-62-1	3.27	0.045	Coding
Igkv4-79	3.39	0.0293	Coding
Mfsd2a	3.49	0.0359	Multiple_Complex
Igkv4-72	3.72	0.0151	Coding
Igkv16-104	3.75	0.009	Coding
Ighv1-19	3.75	0.0477	Coding
Ighv1-36	3.76	0.0359	Coding
Igkv4-55	3.86	0.0102	Coding
Igkv10-95	3.94	0.0233	Coding
Ighv1-85	4	0.0296	Coding
Igkv4-57	4.05	0.0127	Coding
Ighv1-47	4.09	0.0212	Coding
Igkv8-30	4.1	0.0275	Multiple_Complex
Igkv4-71	4.2	0.0161	Coding
Igkv4-70	4.26	0.0137	Coding
Ighv1-5	4.32	0.0167	Coding
Ighv1-66	4.32	0.0301	Coding

Figure S3: Affected genes in the kidney of BC- vs. control-fed mice. Significantly up-regulated genes after 8 days´ BC feeding compared to control feeding (up-regulated > 2.0; < 4.37). Noncoding RNAs and pseudogenes are not included.
